# Growing Weight of OP Evidence: Parathion Linked to Metabolic Effects in Rats

**Published:** 2008-11

**Authors:** Cynthia Washam

Parathion and other organophosphate pesticides, the most widely used class of insecticides, have long been known as neurotoxicants but were only recently linked to metabolic disorders. A new study adds to the growing evidence that parathion may be contributing to epidemics of obesity and diabetes **[*EHP* 116:1456–1462; Lassiter et al.]**.

Obesity and type 2 diabetes have surged in recent decades to the point where two-thirds of U.S. adults are now overweight and approximately 26% have diabetes or prediabetes (elevated fasting blood glucose level below the level considered diabetic). These conditions also are increasing in children, and a growing body of evidence suggests that perinatal exposures to a variety of compounds may have a significant impact on metabolic programming. The authors were prompted to focus on the role of pesticides in these conditions after noting that subpopulations with the highest rates of diabetes and obesity—impoverished residents of inner cities and residents of farming communities—also tended to have the highest pesticide exposures.

The researchers chose parathion as a representative organophosphate. Rats received daily injections of the compound during their first 4 days of life, a developmental period that corresponds to the second to early third trimester in human gestation. Half the treated rats were given a dose (0.1 mg/kg/day) just below the threshold for symptoms of exposure. The other half were given a dose (0.2 mg/kg/day) just above the threshold.

Both doses altered the rats’ metabolism into adulthood, but the effects differed in males and females. Male rats given the lower dose ate about as much as control rats, but outweighed them throughout the 22-week study. Equally important, they showed signs of prediabetes, with elevated fasting serum glucose levels and impaired fat metabolism. High-dose males weighed about as much as controls while consuming less food.

In contrast, both high- and low-dose females weighed less than controls although they consumed at least as much food, indicating a “wasting” condition. This was confirmed by a demonstrated disruption of both glucose and lipid metabolism at both doses.

After reaching adulthood, half the rats were switched to a high-fat diet. Increased fat intake exaggerated parathion’s metabolic effects, particularly in females. The researchers believe early-life exposure to parathion and other chemicals might similarly disrupt human metabolism, thereby contributing to obesity and diabetes. They recommend further studies on the metabolic influence of environmental chemical exposures.

